# “Does It Improve the Mind’s Eye?”: Sensorimotor Simulation in Episodic Event Construction

**DOI:** 10.3389/fpsyg.2019.01403

**Published:** 2019-06-12

**Authors:** Rudy Purkart, Rémy Versace, Guillaume T. Vallet

**Affiliations:** ^1^EA 3082, Laboratoire d’Étude des Mécanismes Cognitifs, Université Lumière Lyon 2, Lyon, France; ^2^CNRS UMR 6024, Laboratoire de Psychologie Sociale et Cognitive, Université Clermont Auvergne, Clermont-Ferrand, France

**Keywords:** event construction, episodic specificity induction, sensorimotor simulation, sensory interference, embodied cognition

## Abstract

Memories are not frozen in the past. Instead, they can be dynamically combined to allow individuals to adapt to the present or even imagine the future. This recombination, called *event construction*, also means that it might be possible to improve memory through specific interventions such as episodic specificity induction (ESI). ESI provides brief training in recollecting the details of a past event that boosts the retrieval of specific details in subsequent tasks if these tasks involve the recombination of memories. However, very little is known about how event construction is accomplished, and this is essential if we are (1) to understand how episodic memory might work and (2) to promote a specific mechanism that will help people remember the past better. The present study assesses the *sensorimotor simulation* hypothesis, which has been proposed within the embodied approaches to cognition. According to these approaches, access to and the recombination of memories occur through the simulation of the sensory and motor propreties of our past experiences. This hypothesis was tested using a sensory interference paradigm. In a first phase, the participants watched videos and then received a specificity or a control induction. In a second phase, they described their memories of the videos while simultaneously viewing an interfering stimulus (dynamic visual noise; DVN) or a gray control screen. In line with a sensorimotor simulation account, the presentation of a DVN during the description of the videos led to a decrease in the number of internal details (details specific to the event) only after the specificity induction rather than the control induction. The findings provide evidence that the specificity induction targets and facilitates the sensorimotor simulation mechanism, thus confirming the crucial involvement of a mechanism of this sort in the constructive functioning of memory.

## Introduction

The human ability to mentally build an event that may have taken place in the past or could take place in the future, and which may involve us personally or involve someone else, is the key process in episodic memory (for a review, see Rubin and Umanath, 2015). The mental construction of detailed events of this sort serves many adaptive purposes by allowing us, for instance, to vividly relive significant episodes of our lives together with the associated emotions or to simulate future events in order to anticipate them or other people’s behavior. These mental constructions appear to rely on the assembly of similar basic materials in the form of small elements of our memories. Nonetheless, the mechanisms involved in event construction remain to be determined. This issue is a major challenge for current memory research in its attempt to understand how we relive our memories, as well as with regard to the development of new methods to improve memory. One promising hypothesis states that *sensorimotor simulation* (Barsalou, 2008, 2009) is the main mechanism permitting the (re)construction of events. The current study thus aims to test whether sensorimotor simulation is required in the event construction process.

For decades, memory was considered to be exclusively oriented toward the past and dedicated to the retrieval of almost intact and exact records of our experiences (i.e., our memories). However, our memories can be distorted (e.g., Gallo, 2006) or merged (e.g., Schacter, 1999), suggesting that memory is more flexible than previously thought. Inspired by the ideas of Bartlett (1932), memory is nowadays conceived of as a dynamic and constructive process that binds together pieces of past experiences rather than as a reproductive process. This constructive nature of memory also makes it possible to “pre-experience,” imagine or simulate events or situations that might happen in the future. In the same way as for memories, these virtual events would seem to be constructed by extracting and recombining pieces of past experiences, in this case to form a novel event that has never actually been experienced (see the *constructive episodic simulation hypothesis*, Schacter and Addis, 2007a,b). Striking cognitive and neural similarities between the construction of past and future events have since been widely demonstrated (for review, see Schacter et al., 2012). From a cognitive standpoint, these two forms of construction are thought to involve a key common process of *event construction* (Romero and Moscovitch, 2012) that consists in assembling and maintaining a mental event by filling it with specific elements of prior experiences (i.e., details related to objects, people, actions). Evidence of the involvement of a common event construction process in the construction of past and future events has recently been provided through the use of an Episodic Specificity Induction (ESI; Madore et al., 2014), a brief intervention designed to selectively improve performance on tasks that may be based on the constructive function of memory.

The ESI is a brief intervention designed to selectively improve performance on tasks that are thought to be based on the event construction process. After viewing a given video clip and completing a filler task, individuals are questioned about their memory of the video clip. This is done using a questionnaire adapted from the cognitive interview (Fisher and Geiselman, 1992; Memon et al., 2010). The cognitive interview is a well-established protocol developed for the legal applications of psychology and is used to interview witnesses using different techniques that are also used in the ESI. Using the ESI questionnaire, the interviewer encourages individuals to close their eyes, to form vivid mental images of specific aspects of the video (e.g., setting, objects, people, actions, etc.) so that they can to focus on them. The participants then describe every detail that comes to mind concerning these aspects without prejudging their relevance and without any interruption from the interviewer. During the interview, the interviewer also asks for certain clarifications about the details mentioned when the individual’s description seems to have reached an end. The logic behind the ESI is that if a task is based, at least in part, on the event construction process, then performance on that task should be impacted and improved by an ESI administered just before the task, as compared to a control induction. Conversely, if a task does not call on event construction then performance should not be impacted by the ESI.

The impact of a prior ESI was studied in two memory and imagination tasks adapted from the autobiographical interview (Levine et al., 2002). The participants viewed pictures of everyday life situations (e.g., cooking a meal, eating in a restaurant, and visiting a museum). For each picture, they were asked to describe, for a period of 3 min and including as many details as possible, a similar and specific situation that had happened to them in the past (memory task) or that could happen to them in the future (imagination task). In line with the autobiographical interview coding procedure, the participants’ verbal descriptions were audio-recorded, transcribed and segmented. Then, each detail was coded as *internal* or *external*. Internal details refer to phenomenological and contextual aspects of the specific event described (i.e., what happened, when it happened, who was present, what the individual felt, what was in the environment). By contrast, external details designate details that do not concern the specific event or that refer to general knowledge about the individual’s life (i.e., habits, retrospective or prospective inferences, peripheral events). The internal details should reflect the extent to which the content of memories (or imaginations) is “episodic,” as defined by Tulving (i.e., the degree to which the events described are accompanied by a sense of reliving and concern a unique experience involving the self; see Tulving, 2002).

Compared to a control induction (i.e., resolving simple math problems), the prior administration of the ESI improved the production of internal details, but not external details, during both the memory and the imagination tasks of the adapted autobiographical interview. Conversely, the prior administration of the ESI had no effect on the production of any types of details in a control picture description task (Madore et al., 2014) in which the participants gave detailed descriptions of complex pictures. As expected, the ESI selectively improved performance on tasks that require event construction (i.e., memory and imagination tasks) and did not affect performance on tasks that do not require event construction (i.e., picture description task). It therefore seems that exposure to the ESI led to increased activity in key brain regions involved in the detailed construction of events in situations where participants imagined future events (Madore et al., 2016). Other studies have provided additional evidence by showing beneficial effects of the ESI on many subsequent tasks thought to require the construction of an event (for a review, see Schacter and Madore, 2016). For instance, the ESI increases the generation of solutions to open-ended social problems (e.g., Madore and Schacter, 2014), the generation of alternative positive outcomes to negative personal future events (Jing et al., 2017), and the generation of unusual uses of common objects (Madore et al., 2015). Overall, these studies have shown that inciting participants to reconstruct a past event in detail in response to the ESI, by encouraging them to focus on specific elements (setting, objects, people, and actions) and to form vivid mental images of these elements, brings about beneficial effects on a variety of tasks. What these tasks share is the need to recombine the same specific elements in order to mentally (re)construct an event, and this is thought to involve the event construction process. It has therefore been suggested that the ESI targets and facilitates the event construction process, which appears to be a key process in memory functioning.

Although it may appear surprising, accurately remembering a past event does not seems to be a necessary prerequisite for observing beneficial ESI effects on subsequent tasks. Recently, the ESI was adapted to produce a version focusing on imagination in which the participants had to imagine a future event instead of remembering details of a previously viewed video (Madore et al., 2018). This version also led to an increase in the production of internal details in memory and imagination tasks, without having any effect on the details generated in a picture description task as in the standard ESI. The authors argue that the ESI effects in both versions come from the *retrieval orientation* bias (Rugg and Wilding, 2000; Morcom and Rugg, 2012; Herron, 2018). Participants would focus their controlled retrieval attempts more on specific elements (setting, objects, people, and actions) during the ESI (Schacter and Madore, 2016). This specific retrieval orientation would then be actively maintained during the subsequent tasks. This bias is thought to facilitate event construction by filling the event with more key elements than are then available for recombination. Nonetheless, this interpretation is not sufficient to explain the event construction process, given that (1) there is no evidence that ESI effects are modulated by an individual’s reserves of cognitive control and (2) we have no indications about the mechanisms involved in event construction.

What is common to the standard and imagination versions of the ESI therefore seems to be the mental simulation of the details of events. In both inductions, individuals are encouraged to form vivid mental images of key elements of the event to be built (setting, objects, people, and actions). With regard to the nature of what is to be simulated, the embodied approaches to cognition make a far-reaching assumption. These approaches suggest that traces of our experiences are grounded in their sensorimotor proprieties (see Versace et al., 2014). In other words, the concept “cat” is solely composed of all our sensory and motor experiences of cats. The grounding of knowledge means that the retrieval of knowledge, or of a given memory, is only possible by mentally simulating the relevant sensory and motor dimensions (see Barsalou, 2008, 2009). This retrieval could occur through the activation of one or more components that may be perceptually present or reactivated. In all cases, the activation can spread to other integrated components of the past event which are not yet perceptually present (Brunel et al., 2009; Vallet et al., 2010) and simulated. Therefore, the mental imagery required by the ESI results from simulations that reach consciousness, even if most simulations remain unconscious (Matheson and Barsalou, 2017). Sensorimotor simulation is therefore the crucial mechanism that enables the (re)construction of our sensorimotor experiences that underlies the recollection or imagination of an event. By asking participants to form vivid mental images of the features of an event, the ESI would generate simulations of many memory trace components, thereby calling greatly on the sensorimotor systems. But how is the ESI supposed to induce greater performance in a subsequent task? It has been shown that transfer between tasks (for instance between the ESI and the subsequent task) benefits from the degree to which the procedures underlying these tasks are similar (for a review, see Kolers and Roediger, 1984). These observations can be related to the notion of transfer-appropriate processing (TAP; Morris et al., 1977) which emphasizes the concordance between the processing involved in tasks. Because the ESI calling greatly on the sensorimotor systems by generating simulations of many memory trace components, a transfer to subsequent tasks that also involve the simulation of specific sensor-motor components should be observed through the facilitation of sensory-motor simulation. In line with this idea, it can be hypothesized that the ESI targets sensorimotor simulation and facilitates future sensorimotor simulations. This could be tested by interfering with the sensorimotor simulation.

Several studies have shown that the most effective method for highlighting the involvement of sensorimotor simulation is to interfere with it (e.g., Brunel et al., 2010; Vallet et al., 2010; Rey et al., 2015, 2017). In one of these studies, participants had to judge whether a picture target represented an artifact (e.g., a violin) or an animal (e.g., a tiger). Each target was previously primed by a sound that could be semantically congruent or not with the picture (e.g., violin sound or car horn for the violin picture). Participants were previously informed that sometimes a colored rectangle (which is actually a visual mask) might be displayed as they hear the sound and were instructed to ignore these stimuli (rectangles and sounds) and to focus on the pictures that they have to categorize. Half of the sound primes were presented simultaneously with a visual mask. It was found a faster processing of semantically congruent items compared to semantically incongruent items. This indicated a cross-modal priming effect; the presentation of a congruent sound prime triggered the automatic simulation of the visual associated component and accelerated the decision task compared to the presentation of an incongruent sound prime. More interestingly, the visual mask interfered with the priming effect and slowed down the decision task only in the congruent condition, which excludes a potential attention effect (Vallet et al., 2013). To test the hypothesis put forward in the current study, one appropriate approach would be to interfere with the sensorimotor simulation by simultaneously presenting a visual mask during the (re)construction of an event after the prior administration of an ESI.

However, we do not all have the same capacity to generate and use mental imagery. For instance, individuals with vivid visual mental images have been found to have phenomenologically richer memories (D’Argembeau and Van der Linden, 2006). By contrast, individuals with poor visual mental images have been found to have a reduced sense of reliving their memories (Greenberg and Knowlton, 2014). Directly related to event construction, a useful distinction has been made between the ability to imagine the visual proprieties of objects (object imagery) and the ability to imagine spatial relations (spatial imagery) (Blajenkova et al., 2006). Using this distinction, the detrimental effect of a dynamic visual noise (DVN; McConnell and Quinn, 2004) combined with imagery during the recall of detailed videos has been found to be related to spatial imagery (Sheldon et al., 2016). This result implies that individuals differ in how they simulate some aspects of past events and how they are affected by an imagery or sensory interference, and that the simulation of spatial information plays a leading role in event construction. Because the current study aims to investigate the involvement of sensorimotor simulation in the beneficial effects of the ESI on event construction in a sensory interference paradigm, it was necessary to control for the participant’s visual imagery abilities.

The constructive nature of memory has been highlighted in studies that have used an ESI to selectively improve performance in memory and imagination tasks (Schacter and Madore, 2016). However, the core mechanism underlying the event construction process remains to be specified. According to the embodied approaches, this mechanism should be sensorimotor simulation (see Barsalou, 2008, 2009; Versace et al., 2009, 2014). The aim of the present study is to assess whether this sensorimotor simulation is required in the event construction process. This could be done by observing the impact of sensory interference on the ESI effect during descriptions of a remembered event. More specifically, if the ESI targets and facilitates sensorimotor simulation as expected, then presenting an interfering sensory stimulus to disrupt the simulation of visual details during descriptions of remembered events should reduce the quantity of internal details produced during the description of events following the ESI and cancel out the benefits conferred by the ESI.

In the current study, the participants had to encode non-auditory videos (encoding phase) before receiving an ESI or a control induction (induction phase). Immediately afterward, they had to describe the videos presented in the encoding phase in as much detail as possible (description task). During the description task, a DVN was simultaneously displayed for half of the description trials, while a control stimulus (a gray screen) was displayed for the other half of the trials. The DVN consisted of a matrix of small, moving, black, and white squares that formed a constantly changing pattern that is thought to passively occupy visuo-perceptual imagery processes without the involvement of executive or attentional resources (Quinn and McConnell, 1996). For instance, DVN did not influence performances on tasks that does not require the use of imagery such as a number comparison task contrary to a task that require to make size comparisons between the names of animals presented verbally (Dean et al., 2005). The simultaneous presentation of the DVN was expected to disrupt the simulation of visual details. In line with the above-mentioned hypothesis, it was expected that the presentation of DVN during the description task (compared to control stimuli) would generate a greater interference effect on the production of internal details after the ESI than after the control induction.

## Materials and Methods

### Participants

Thirty-five young adults (*M_age_* = 21.5, *SD_age_* = 2.03, 28 female) took part in this study. They were recruited at Lyon 2 University. All the participants had normal or corrected-to-normal vision and audition, and no history of neurological or psychiatric disorders. All the participants were French native speakers.

This study was carried out in accordance with the recommendations of the French Law (Loi Jardé n°2012-300) with written informed consent being obtained from all the subjects in accordance with the Declaration of Helsinki. An ethics approval was not required for the current study as per applicable institutional and national guidelines.

### Materials

#### OSIQ

The Object-Spatial Imagery Questionnaire (OSIQ; Blajenkova et al., 2006; French version by Léo Dutriaux, unpublished) assesses the ability to imagine an object’s shape, texture and color (object imagery score), and the ability to imagine location, movements, and spatial relationships (spatial imagery score). For each score, the participants are asked to answer 15 questions about their use of imagery in real-life situations (e.g., for the object score: “I can close my eyes and easily picture a scene that I have experienced.”; for the spatial score: “I can easily imagine and mentally rotate 3-dimensional geometric figures.”). Each question is rated on a 5-point Likert scale (1 = totally disagree to 5 = totally agree).

#### Induction Videos

Two videos of 5 min each were used during the induction phase and took the form of excerpts from French theater plays collected on the video-sharing website YouTube^1^. Each of them was accompanied by sound and depicted six adults (three females) engaged in lively discussions in a restaurant or in a living room. The videos were chosen based on the richness of the setting and the amount of action and dialogue.

#### Episodic Specificity Induction (ESI)

The original version proposed by Madore et al. (2014) was recently translated into French and validated by our team (Purkart et al., 2019). This induction is based on the cognitive interview (Fisher and Geiselman, 1992) and guides the interviewer in probing deeply into the participant’s memory of an event or a recently viewed video. Participants are asked to describe the setting of the video, the people present, and the actions performed. They are asked to close their eyes and to generate images in their minds, and then to report everything they remember in as much detail as possible. At the end of the description of each element (setting, people, and action), the interviewer requests a more detailed description of some of the elements mentioned. (For the full protocol in English, see Madore et al., 2014).

#### Control Induction (CI)

The control induction consisted in asking the participants to rank series of digits in ascending order. This task was selected because it does not require the participants to remember anything while still actively occupying them. The control induction and the specificity induction had the same length.

#### Event Videos–Stimuli and Titles

Twenty short audio-free video-clips that depicted real-world events (10–20 s) were selected from the videos used by Sheldon et al. (2016). Four videos had a mix of males/females, seven had only female characters, and nine had only male characters. Nine were filmed outdoors, while 11 were filmed indoors. The videos were divided into two equivalent sets which were randomized across participants and inductions in order to avoid a description bias generated by the characteristics of the videos. For each video, a short title was created and written on a slide. The title was also distinctly pronounced by an artificial female voice and audio-recorded (French female voice Audrey, Text to Speech in macOS).

#### Dynamic Visual Noise (DVN)

Five different 5-min DVN clips were created using the available source code provided by McConnell and Quinn (2004) ^2^ in the same way as was done by Sheldon et al. (2016). The DVN clips consisted of a matrix of randomly moving black and white squares. This created a continuously changing pattern that passively occupied the participants’ visuo-spatial processes and interfered with their visual mental imagery. The DVNs were presented on a 21.5-inch computer screen (1920 × 1080 pixels).

#### Non-interfering Control Stimulus

A non-interfering control stimulus was created and consisted of a gray screen (Hex color code #CCCCCC). This was presented on a 21.5-inch computer screen (1920 × 1080 pixels).

#### Experimental Settings

The experiment was coded using the OpenSesame software (Mathôt et al., 2012). The computer used was an Apple MacBook Pro (2.8 GHz Intel Core i7; Radeon Pro 555 2048 Mo; 16 Go 2133 MHz LPDDR3; 15-inch, 2017) connected to an external screen (Acer ET221Q; 21.5-inch; 1920 × 1080 pixels). This was placed on an adjustable platform so that the center of the screen was at the same height as the participants’ heads and was positioned 60 cm from their noses. Sennheiser HD206 headphones were used because these provide excellent insulation against ambient noise. The voice recorder used to record the descriptions was an Olympus WS-852.

### Procedure

**FIGURE 1:**
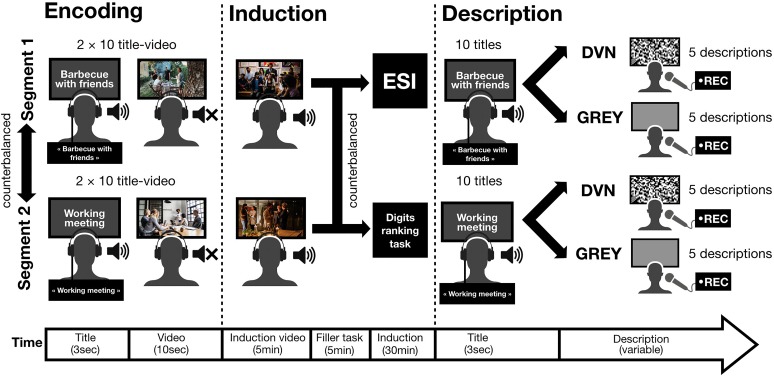
The experiment was administered in two segments with an interval of 5 min between them and consisted of three phases. At encoding, 10 title-video associations were presented twice in succession. At induction, the participants watched a video-clip, completed a filler task, and then received the specificity induction (ESI) or the control task (digit ranking task). At description time, the titles were displayed on the screen and the participants had to describe the associated videos while watching the dynamic visual noise (DVN) or the control stimulus. Photos are for illustrative purposes only and have been downloaded from the photography sharing website unsplash.com. These photos are used in accordance with the Terms and Conditions of the website which do not require further permission.

The participants were placed in a dark room in front (≈60 cm) of a computer display raised to face height to ensure that their visual field would be entirely occupied by the display. They wore headphones that broadcast the audio stimuli and they were comfortably seated. The sound level was first adjusted by the participant using a test track. The participants adjusted the sound so that they could clearly hear what the voice on the test track was saying. Before the beginning of the test, they were informed that they were taking part in a study about the way in which individuals explore their memories. The test was administered in two segments, with an interval of 5 min between them, and with each consisting of three phases (Figure 1). Both segments were completed by each participant in a within-subject design.

(1) Encoding phase – During the encoding phase, the participants were informed that they would see several short video-clips with titles and were instructed to pay close attention to the details of each video because they would be asked to remember them later. After the instructions, a set of ten titles was presented, each followed immediately by the associated video. Each title-video association was presented twice successively to maximize encoding. Sets of videos were randomized across participants and segments.
(2) Induction phase – After the encoding phase, the participants were informed that they would see a longer video and were instructed to pay close attention to it because its content might be discussed shortly after viewing. The participants watched one of the two induction videos, completed a filler task and, finally, were either interviewed about the video using the ESI script or completed the control task (digit ranking task). The video-induction pairing and the induction type (ESI or control) was counterbalanced across participants and segments.
(3) Description phase – After the induction phase, the participants were informed that each video title in the encoding phase would be replayed and that their memory about the details of the related video would be tested. The participants were asked to respond to each title by describing, in as much detail as possible, their memory of the event depicted by the video associated with the title. They were asked to press the space bar once their description was finished. During the description, the participants simultaneously viewed either the DVN stimulus (interference condition) or the control screen (control condition). Half of the descriptions were produced during the presentation of the DVN. The DVN and control stimuli were randomized across videos and participants. The descriptions were audio-recorded and then transcribed by the experimenter. The participants were instructed to look at the center of the screen and try to blink as little as possible throughout this phase. The experimenter was sitting next to the participant and outside of its visual field to discreetly control that the participant complies with this instruction.

After the description phase, the participants were asked to contribute further to the study to make it possible to obtain a satisfactory volume of data. They started the second segment of the experiment after a 5-min break during which they completed a filler task that consisted in positioning several series of arrows end-to-end on a grid from a given starting point (e.g., →↑→↓↓←). This filler task was proposed to ensure that sufficient amount of time (in which the participants’ memory is not solicited) has passed before the beginning of the next segment to counter a carryover effect from the first segment. Previous studies have shown that a 5-min break is sufficient to observe an induction effect without carryover effect (e.g., Madore and Schacter, 2016). The whole experiment lasted between 1.5 and 2 h.

#### Coding

The descriptions were audio-recorded, transcribed, and coded for internal and external details according to the Autobiographical Interview procedure (Levine et al., 2002). Internal details refer to phenomenological and contextual aspects of the video described (i.e., what happened, who was present, what was in the environment). External details refer to detail that does not concern the content of the video described (i.e., inferences, posteriors judgments, information unrelated to the video or off-topic). All the transcriptions were coded by a trained rater, while a second trained rater coded a random selection (15%) of the descriptions in order to assess the reliability of coding, which was acceptable (Cronbach’s alpha = 0.81 for internal details and 0.89 for external details). For more information about the coding procedure, see Appendix A. Neither of the raters knew which induction had been administrated before the descriptions they coded, and which visual stimuli (DVN or control stimulus) had been displayed.

## Results

The mean number of internal and external details was computed for each experimental condition. Two separate repeated-measures analyses of covariance (ANCOVA) were performed on the type of details with Induction (ESI vs. Control) and Interference (DVN vs. Control) as within-subject factors, and object and spatial scores of the OSIQ as covariates of interest. As in Sheldon’s et al. 2016 study, these covariates were considered because the interference effect of DVN is likely to be modulated by imagery abilities. A pairwise comparison for the interaction was performed using Fisher’s Least Significant Difference Test because less than 4 comparisons were made. The threshold of statistical significance for the ANCOVA was *p* < 0.05. The threshold of statistical significance for the pairwise comparison was corrected with the Holm–Bonferroni method (Holm, 1979). Table 1 displays descriptive statistics.

**Table 1:** Descriptive statistics for internal and external details on the description task as a function of interference and induction.

INTERFERENCE	INDUCTION	Internal		External	
CONTROL	CONTROL	19.62	(8.23)	0.76	(0.84)
	ESI	21.91	(8.51)	0.87	(0.84)
DVN	CONTROL	19.50	(8.72)	0.85	(0.82)
	ESI	21.05	(9.09)	0.90	(0.82)

*Numeric values are presented as mean per trial (with standard deviation in parentheses).*

### External Details

The analysis revealed that the two-way Induction × Interference interaction for external details was not significant as well as the main effects of Induction and Interference (*Fs* < 1).

### Internal Details

**FIGURE 2:**
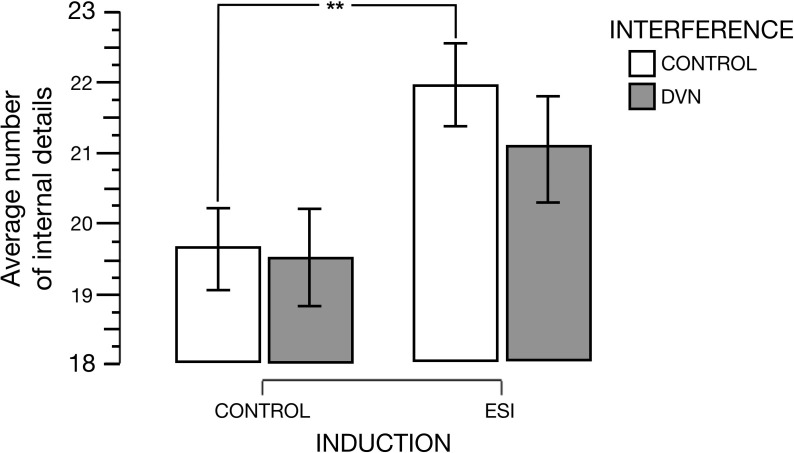
The average number of internal details produced for the description task as a function of the induction (specificity induction or ESI vs. control induction) and of the interference stimuli (dynamic visual noise – DVN vs. control), with object and spatial scores on the Object-Spatial Imagery Questionnaire being considered as covariates. Vertical bars represent standard errors of the means. Significant results are denoted by two asterisks (*p* < 0.01).

The analysis revealed that the two-way Induction × Interference interaction for internal details was significant with a medium effect-size [*F*(1,32) = 5.669, *p* = 0.023, η^2^ = 0.12] (see Figure 2), but that the main effects of Induction and Interference were not (*F* < 1). Decomposition of the interaction was made by observing (1) whether the effect of the ESI is significant as expected when a non-interfering control stimulus is displayed during the description (i.e., whether the number of internal details produced during the description task is greater after the ESI than after the control induction in the interference control condition); and (2) whether the effect of the ESI remain significant or not when a DVN is displayed during the description. These two comparisons were computed using *t*-tests corrected with the Holm–Bonferroni method (Holm, 1979). The tests showed that in the control interference condition, the participants produced significantly more internal details after the ESI (*M* = 21.96, *SD* = 8.51) than after the control induction [*M* = 19.62, *SD* = 8.23; *t*(34) = 2.588; *p* = 0.007; *d* = 0.437; corrected significant threshold of 0.025] with a medium effect-size, and this difference was no longer significant in the DVN interference condition [*t*(34) = 1.329; *p* = 0.096; *d* = 0.225; corrected significant threshold of 0.05]. The average number of internal details produced in each interference condition did not differ significantly after the control induction (*p* > 0.05) as well as after the ESI (*p* > 0.05).

### Covariates

The analysis revealed that the three-way Induction × Interference × Object Imagery interaction for internal details was not significant [*F*(1,32) = 2.534, *p* = 0.121, η^2^ = 0.05] with a modest effect-size, but the three-way Induction × Interference × Spatial Imagery interaction for internal details was significant [*F*(1,32) = 6.013, *p* = 0.020, η^2^ = 0.130] with a medium effect-size. The two-way Induction × Object Imagery interaction for internal details was not significant (*F* < 1), as well as the two-way Induction × Spatial Imagery interaction for internal details (*F* < 1). Identically, the two-way Interference × Object Imagery interaction for internal details was not significant (*F* < 1), as well as the two-way Interference × Spatial Imagery interaction for internal details (*F* < 1). The analysis also revealed that no interaction with Object or Spatial Imagery was significant for external details (*F* < 1).

### Carryover

A repeated-measures ANOVA was conducted with the induction order as an independent variable to determine whether the number of internal details produced on the description task differed as function of whether participants received the ESI in the first segment or in the second segment. The analysis revealed that the two-way Induction × Order interaction for internal detail was not significant [*F*(1,31) = 1.308, *p* = 0.262]. This indicates that results are unlikely attributable to carryover effects.

## Discussion

A major conceptual shift that has occurred during the past two decades lies in the fact that memory is no longer considered as a static system, but instead as a dynamic and constructive process. As such, memory makes it possible to construct past, future or imagined events by recombining pieces of our past experiences (see the *constructive episodic simulation hypothesis*, Schacter and Addis, 2007a,b). The event construction process is illustrated by the beneficial effect of the ESI (e.g., Madore et al., 2014). However, the mechanism driving event construction remains to be determined. This is an important gap in our knowledge that considerably limits our understanding of the constructive functioning of memory and prevents the development of efficient interventions designed to improve this process. A promising hypothesis within the embodied cognition framework states that our experiences are grounded in their sensorimotor components (Versace et al., 2014). Accordingly, each sensorimotor components of a given memory trace can be dynamically and automatically reactivated and simulated through a *sensorimotor simulation* mechanism (Barsalou, 2008, 2009). This sensorimotor simulation is thought to permit the (re)construction of past or imagined events and therefore to be the core mechanism involved in the effect of the ESI. The present study tested this hypothesis using a visual interference paradigm.

After a classic ESI or control induction, the participants had to describe videos while seeing a dynamic visual mask (DVN) or a control stimulus. In the DVN condition, they reported a smaller number of internal details only following the ESI but not the control induction. In the control interference condition, the participants produced significantly more internal details after the ESI than after the control induction, as had been expected (see Madore et al., 2014). Conversely, the difference between the types of induction was no longer significant in the DVN interference condition. Therefore, the simultaneous presentation of a DVN during the descriptions of remembered videos significantly impaired the benefits generated by the prior administration of the ESI.

According to the embodied approach to memory (e.g., Versace et al., 2014; Barsalou, 2015), the display of a video preceded by a title during the encoding phase activated the participant’s neurons implicated in the perception of the sensorimotor components of the present situation. These neurons formed a specific pattern and were associated to each other to form a memory trace of the event. During the description phase, the presentation of a previously encoded title (the cue) should automatically trigger the reactivation/simulation of the other associated components of the memory trace (see Rey et al., 2017). Many studies have previously evidenced the functioning of this mechanism in a robust way (for a review, see Dijkstra and Post, 2015). These simulations allow to recreate the state of the sensorimotor systems as they were when the video was watched, and by so to reconstruct this specific experience. Among these simulations, those that have reached consciousness then generated mental imagery (Matheson and Barsalou, 2017) that should underpin the subsequent description of the video and the production of internal details. When the description phase was preceded by the ESI, the resulting facilitation effect on sensorimotor simulation should have resulted in the simulation of a larger number of components of the memory trace corresponding to the encoded video, and in the production of a larger number of internal details. Because perception and memory may make use of the same sensorimotor system (Riou et al., 2011; Rey et al., 2015), the simultaneous presentation of a DVN in the participant’s visual field and its passive processing during the description should have solicited the sensorimotor system needed for simulations. This would have disrupted and reduced the facilitation effect generated by the ESI on the sensorimotor simulation.

The absence of an interference effect in the control induction condition supports this interpretation and argues against the possibility that the interference effect generated by the DVN was due to attentional causes. Indeed, an attentional effect should have been found independently of the type of induction. No attentional effect has been found in the past by authors who have used DVN (e.g., Parker and Dagnall, 2009; Perfect et al., 2012; Sheldon et al., 2016). Furthermore, the masking paradigm has already been shown to be dependent on this sensorimotor simulation and not on an attentional effect (Vallet et al., 2013). Moreover, the results of the current study argue against the hypothesis that ESI effects are strategic in nature and, consequently, against the retrieval orientation account (e.g., Madore et al., 2018). Indeed, there is no reason why the beneficial effects of the ESI should disappear only when the DVN was displayed on the screen. A bias in retrieval orientation should have persisted for all the trials that follow the ESI independently of the type of interference (DVN vs. control). Since the DVN did not tax the participants’ cognitive control resources (see Quinn and McConnell, 1996), the present results support the hypothesis that ESI targets and facilitates the sensorimotor simulation mechanism rather than simply biasing retrieval orientation.

The finding that an imagination or a memory specificity induction (standard ESI vs. imagination version of the ESI) produce similar beneficial effects on the description of past or imagined events (Madore et al., 2018) provides additional arguments in favor of the sensorimotor simulation hypothesis. Indeed, as the imagination version does not require the retrieval of a specific past event, remembering does not appear to be a prerequisite for observing an induction effect. On the contrary, both versions of the ESI require the simulation of many sensorimotor components of either past experiences or imagined ones. Both versions involve the detailed description of the event and the formation of vivid mental images, which both result from the sensorimotor simulation of the components of memory traces of specific past experiences. The ESI would boost sensorimotor simulation, which would, in turn, improve performance on all subsequent tasks that require the simulation of sensorimotor components. Logically, the induction should not have any effect on tasks that do not require this specific kind of simulation, such as the picture description task. For instance, this description task only requires participants to describe what they see in a complex picture (e.g., a messy desk). This dissociation of performances is predicted by specific embodied memory models such as the Activation-Integration model (Act-In; Versace et al., 2014).

If the benefits generated by the ESI are related to a facilitation of sensorimotor simulation, it remains to be determined whether the sensorimotor simulation mechanism can be trained to produce a longer-lasting facilitating effect. Indeed, the benefits generated by the ESI seem to be transitory, considering that no carryover effect from the ESI has been found in previous studies (e.g., Madore et al., 2014; Madore and Schacter, 2014, 2016). Some cognitive interventions have been designed to train and improve autobiographical recall (i.e., recall of personal events) and have shown long-term benefits. Overall, these interventions share many characteristics: they (a) take place over multiple sessions, (b) include psychoeducation (e.g., Neshat-Doost et al., 2013), (c) encourage individuals to produce specific memories related to different times or emotions (e.g., Raes et al., 2009; Ricarte et al., 2012) and to discuss them in groups (e.g., Blairy et al., 2008), and (d) encourage individuals to form vivid mental images and to imagine future events (e.g., Belleville et al., 2006; Ernst et al., 2015). Despite these studies, it is not clear whether these long-term effects are due to better learning retrieval strategies or to improved memory functioning *per se*. Despite this, all the above-mentioned interventions could be united by the fact that that they encourage individuals to frequently simulate components of specific past experiences. Further investigations are needed to provide convincing evidence in favor of a long-term facilitation of the sensorimotor simulation mechanism. Such studies would have substantial clinical implications with regard to the development of interventions designed to improve the memory functioning of individuals who are characterized by memory losses concerning their past.

Some potential limitations must be considered. A first concern is that participants could have guessed that the DVN was supposed to disturb them and they could have showed compliance. But the average number of internal details produced for the description task after a control induction was not statistically different under the two interference conditions (DVN and control), contrary to what would have been expected in a case of compliance. A second concern is that participants could have guessed that the ESI would boost their performance in the description task compared to the control induction. Yet, at the end of the experiment and during the debriefing, in which the purpose of the study was explained to the participants, none of them reported that they had guessed the aim of the study or the expected effect of the specificity induction. Finally, no carryover effects were found in the present study. Given the aforementioned elements, it is very unlikely that participants had guessed and complied with the study’s main hypothesis regarding the interaction between the type of interference and the type of induction.

Another concern is that no effects were found in the present study regarding the production of external details. Using tasks adapted from the autobiographical interview (Levine et al., 2002), some previous studies have found no effect of the ESI on the production of external details (e.g., Madore et al., 2014), while some studies have shown that the ESI reduces this production (e.g., Madore et al., 2018). Because the autobiographical interview involves participants reflecting on elements of their own lives, this task is likely to generate the production of external details that concern irrelevant or repetitive autobiographical events, in parallel with the production of internal details that concern the autobiographical event mainly narrated. In some cases, the ESI can help reduce the production of external details in favor of an increase in the production of internal details. In the case of the present study, the description task involves describing a short event that does not involve the participant personally, which considerably limits the production of external details (often limited to inferences). The production of external details is probably too low (the average number of external details produced is less than 1) to observe an effect of the ESI.

The originality of the current study was to directly assess what mechanism(s) may drive the event construction process. We provide additional evidence in favor of the constructive conception of memory defended by the *constructive episodic simulation hypothesis* (Schacter and Addis, 2007a,b) by taking the investigation of ESI effects further by means of an embodied approach to memory. These approaches relate to one another because both of them consider that memories are being reconstructed rather than retrieved intact. But the idea carried by the embodied approach that sensorimotor simulation is one of the main mechanisms underlying this reconstruction brings new hypotheses concerning the ESI effects. By using a sensory interference paradigm, we showed that the ESI appears to target and to facilitate the sensorimotor simulation mechanism, thus confirming the crucial involvement of this mechanism in the constructive functioning of memory. The embodied approaches to cognition thus offer an interesting research avenue, allowing us to gain a better understanding of how event construction might operate. This framework could also be of interest in the search for further clarifications relating, for example, to the durability or amplification of ESI effects.

## Ethics Statement

This study was carried out in accordance with the recommendations of the French Law (Loi Jardé n°2012-300) with written informed consent being obtained from all the subjects in accordance with the Declaration of Helsinki. An ethics approval was not required for the current study as per applicable institutional and national guidelines.

## Author Contributions

RP participated in all the aspects of the study (design of the experiments, conduct of the experiments, analysis and interpretation of the data, and redaction of the manuscript). RV and GV participated in the design of the experiments, interpretation of the data, and redaction of the manuscript.

## Conflict of Interest Statement

The authors declare that the research was conducted in the absence of any commercial or financial relationships that could be construed as a potential conflict of interest.

## Footnotes

1 www.youtube.com

2 https://www.st-andrews.ac.uk/

## Appendix

**Appendix A:** Description of the coding procedure applied in the study and derived from Levine et al. (2002).

When, for example, the task requires the participant to recount a specific event which must not last for more than a few hours or take place in more than one location (e.g., birthday party): a *specific* detail is a detail that is specific to the main event that is recounted (i.e., that only relates to this precise event) and that relates primarily to the episodic, phenomenological and spatiotemporal aspects of the event. A *specific* detail is characterized by a proposition consisting of at least a subject and a verb, and, complementarily, also an object or an attribute (e.g., “Peter dropped his sandwich” = 1 detail). If the subject is cited for the first time then this counts as an additional specific detail (e.g., “Peter” = 1 detail). If the object (1) is a direct or indirect object; (2) is a noun or noun phrase designating an individual or group of individuals (e.g., “Peter greeted Paul”); (3) is cited for the first time, then this object is counted as an additional detail (e.g., “Paul” = 1 detail). If there is an adverb or other phrase or clause describing the time (e.g., “*Yesterday*, I went to the swimming pool.”), place (e.g., “I swam several lengths *in the outdoor pool*.”), manner (e.g., “He coughed *noisily*.”), means (e.g., “She nailed the planks *with a hammer*.”), goal (e.g., “I wrapped myself up *to avoid the cold*.”), cause (e.g., “Peter was punished *because of his brother*.”), consequence (e.g., “I screamed so much *I lost my voice*.”), or quantity (e.g., “This DVD cost *twenty euros*”), then it is counted as an additional specific detail. Even though these various descriptive elements are not essential, they provide relevant additional information relating both to the circumstances of the described action and the phenomenological or spatio-temporal details.

A *non-specific* detail is a detail that is not specific to the main event recounted or that is common to multiple similar events (e.g., “Every morning, I go for a run.”). This type of detail can also take the form of an inference (e.g., “They must have argued when they arrived.”), a retrospective reflection (e.g., “It was a moment that left its mark on me.”), a reiteration (e.g., “So Peter greeted Paul, and…”), a piece of general knowledge (e.g., “Paris is the capital of France.”) or information about oneself (e.g., “I often go to restaurants.”). The rating method for this type of detail is identical to that used for specific details.
